# Supplementary material for: Assessing the real implications for CO2 as generation from renewables increases
Source: Nat Commun. 2025 Aug 2;16:7124. doi: 10.1038/s41467-025-59800-4 (PMC12318073; doi:10.1038/s41467-025-59800-4)
Supplement: Supplementary file 1 — Supplementary Information [file 41467_2025_59800_MOESM1_ESM.pdf]

Assessing the real implications for CO_2 as generation from renewables increases

Dhruv Suri[1], Jacques de Chalendar[1], Inês M. L. Azevedo[1,2,3,4,5]

[1]Department of Energy Science & Engineering, Doerr School of Sustainability, Stanford University, California, United States
[2]Precourt Institute for Energy, Doerr School of Sustainability, Stanford University, California, United States
[3]Woods Institute for the Environment, Doerr School of Sustainability, Stanford University, California, United States
[4]Civil and Environmental Engineering, School of Engineering, Stanford University, California, United States
[5]Visiting Professor, Nova Business School, Portugal

Supplementary Information

Contents

Supplementary Figures 2
Supplementary Figure 1: Pearson correlation coefficients between exogenous variables and thermal power plant metrics . . . 2
Supplementary Figure 2: Distribution of the Durbin-Watson (DW) test statistic . . . 3
Supplementary Figure 3: Emissions intensity vs. capacity factor (CAISO Natural Gas 1) . . . 4
Supplementary Figure 4: Emissions intensity vs. capacity factor (CAISO Natural Gas 2) . . . 5
Supplementary Figure 5: Emissions intensity vs. capacity factor (CAISO Natural Gas 3) . . . 6
Supplementary Figure 6: Capacity factor vs. emissions intensity (CAISO Natural Gas 4) . . . 7
Supplementary Figure 7: Emissions intensity vs. capacity factor (ERCOT Natural Gas 1) . . . 8
Supplementary Figure 8: Emissions intensity vs. capacity factor (ERCOT Natural Gas 2) . . . 9
Supplementary Figure 9: Capacity factor vs. emissions intensity (ERCOT Natural Gas 3) . . . 10
Supplementary Figure 10: Emissions intensity vs. capacity factor (ERCOT Natural Gas 4) . . . 11
Supplementary Figure 11: Emissions intensity vs. capacity factor (ERCOT Coal) . . . 12
Supplementary Figure 12: Hourly variation in emissions, generation, and emissions intensity (2018–2023) . . . 13
Supplementary Figure 13: Change in CO_2 emissions intensity of thermal power plants under increasing levels of renewable generation . . . 14

Supplementary Tables 15
Supplementary Table 1: Panel regression coefficients for natural gas plants in CAISO . . . 15
Supplementary Table 2: Panel regression coefficients for natural gas plants in ERCOT . . . 15

Supplementary Note 1 16

Supplementary Figures

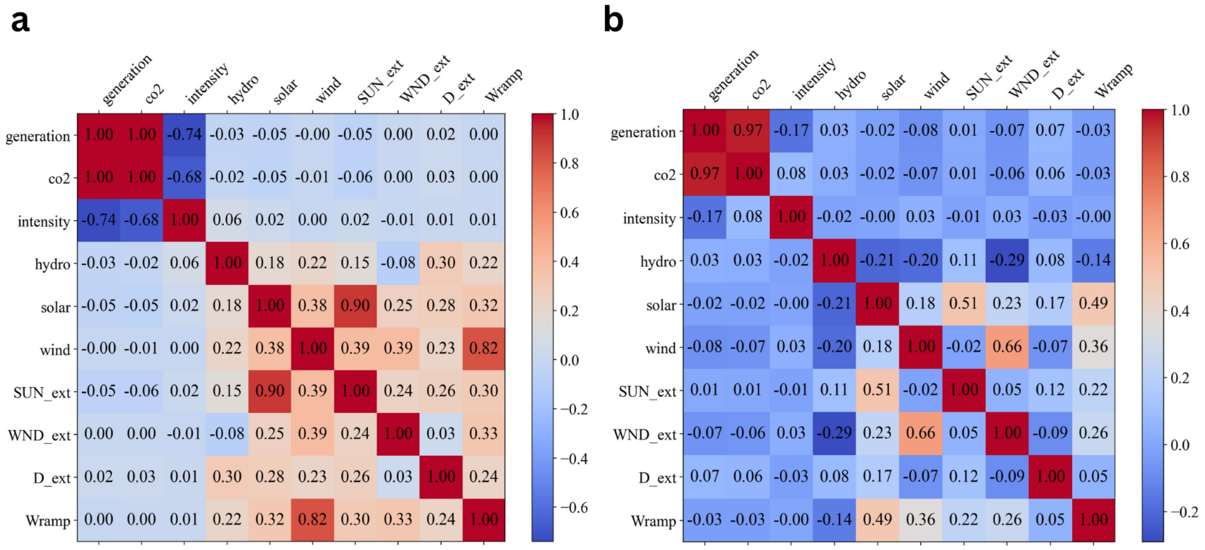

Supplementary Figure 1: Pearson correlation coefficients between exogenous variables and thermal power plant metrics in two U.S. electricity markets. (a) California Independent System Operator (CAISO) and (b) Electric Reliability Council of Texas (ERCOT). Each heatmap represents the pairwise Pearson correlation coefficients between key operational and environmental variables, including thermal power generation, CO_2 emissions, emissions intensity, hydroelectric generation, solar generation, and wind generation. The Pearson correlation coefficient ranges from -1 to 1, where values near 1 indicate strong positive correlation, values near -1 indicate strong negative correlation, and values close to 0 suggest no linear relationship. The color scale reflects the strength and direction of correlations, with red indicating strong positive correlations and blue indicating strong negative correlations. In both markets, renewable generation (solar and wind) shows moderate correlations with external generation from neighboring regions, suggesting some degree of coordinated electricity market interactions. However, the correlation between renewable generation and emissions intensity varies, reflecting regional differences in grid dispatch dynamics, fuel mix composition, and thermal plant flexibility. These correlations provide insights into the potential impact of renewable integration on thermal plant operations and emissions.

a **b**

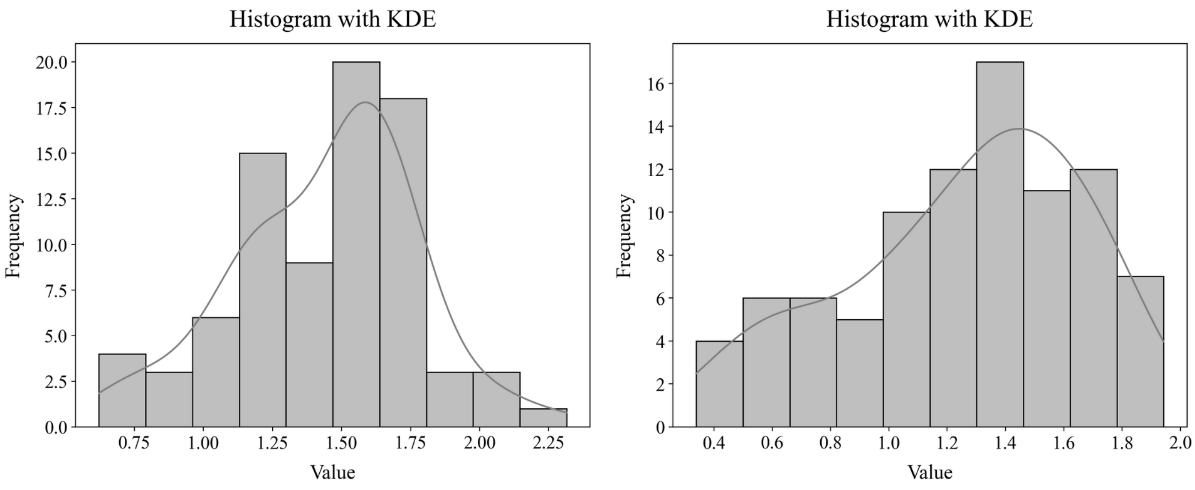

Supplementary Figure 2: Distribution of the Durbin-Watson (DW) test statistic for assessing autocorrelation in residuals from panel regression models. (a) California Independent System Operator (CAISO) and (b) Electric Reliability Council of Texas (ERCOT). Each histogram represents the empirical distribution of DW test statistics across thermal power plants, with an overlaid kernel density estimate (KDE) to illustrate the smoothed probability density function. The Durbin-Watson test evaluates the presence of first-order serial correlation in regression residuals, with values close to 2 indicating no significant autocorrelation, values approaching 0 suggesting strong positive autocorrelation, and values near 4 indicating strong negative autocorrelation. The observed distributions indicate that approximately 73% of thermal power plants exhibit DW statistics between 1 and 2, suggesting limited serial correlation in the model residuals. This result supports the assumption that, for most plants, the regression residuals do not exhibit significant time-dependent structure, which would otherwise require additional correction through methods such as generalized least squares (GLS) or Newey-West standard errors.

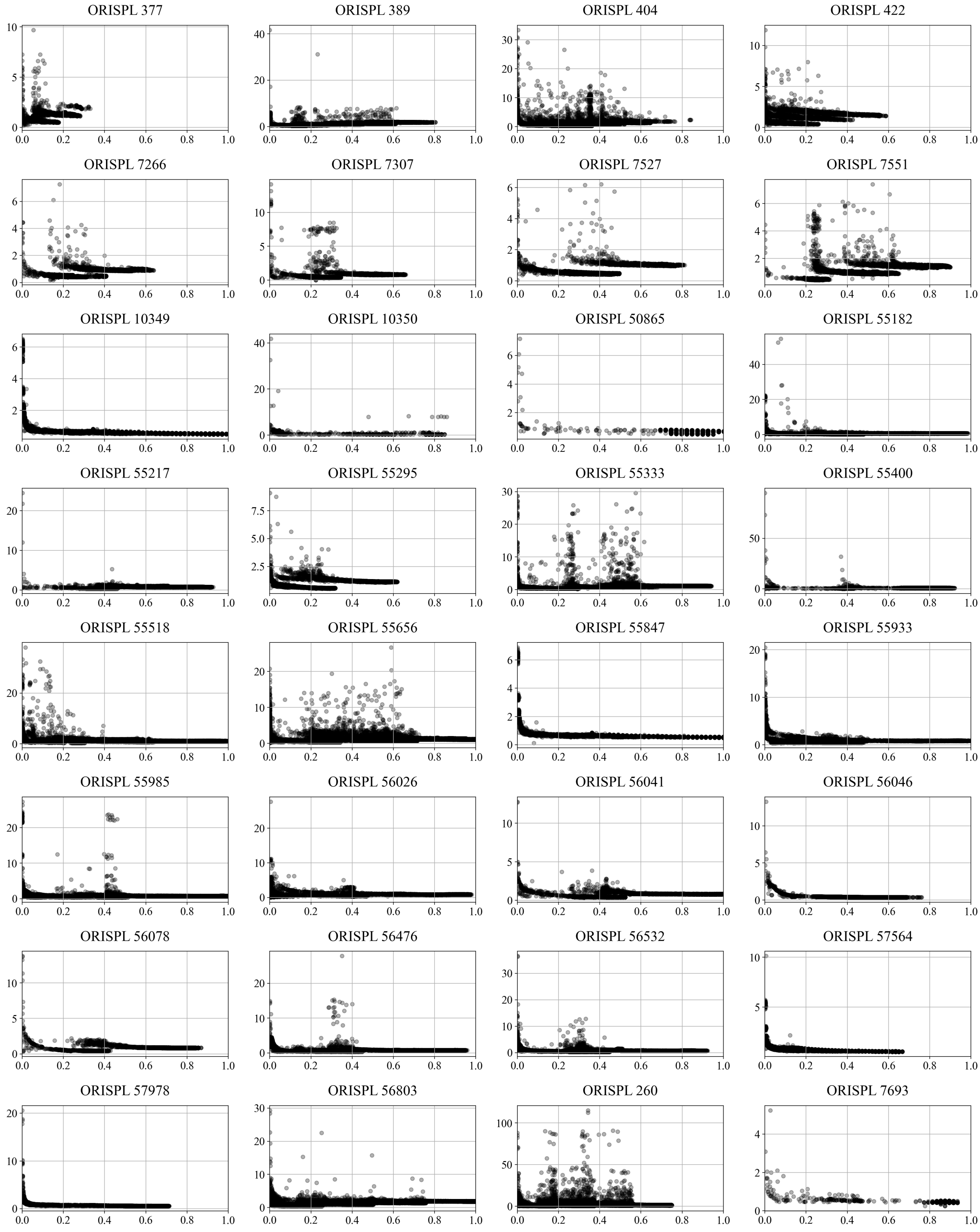

Supplementary Figure 3: Relationship between emissions intensity (tons of CO_2 per megawatt-hour) and capacity factor for natural gas power plants in the California Independent System Operator (CAISO) region. Each plant is identified by its unique Office of Regulatory Information Systems Plant Location (ORISPL) code, assigned by the U.S. Energy Information Administration (EIA).

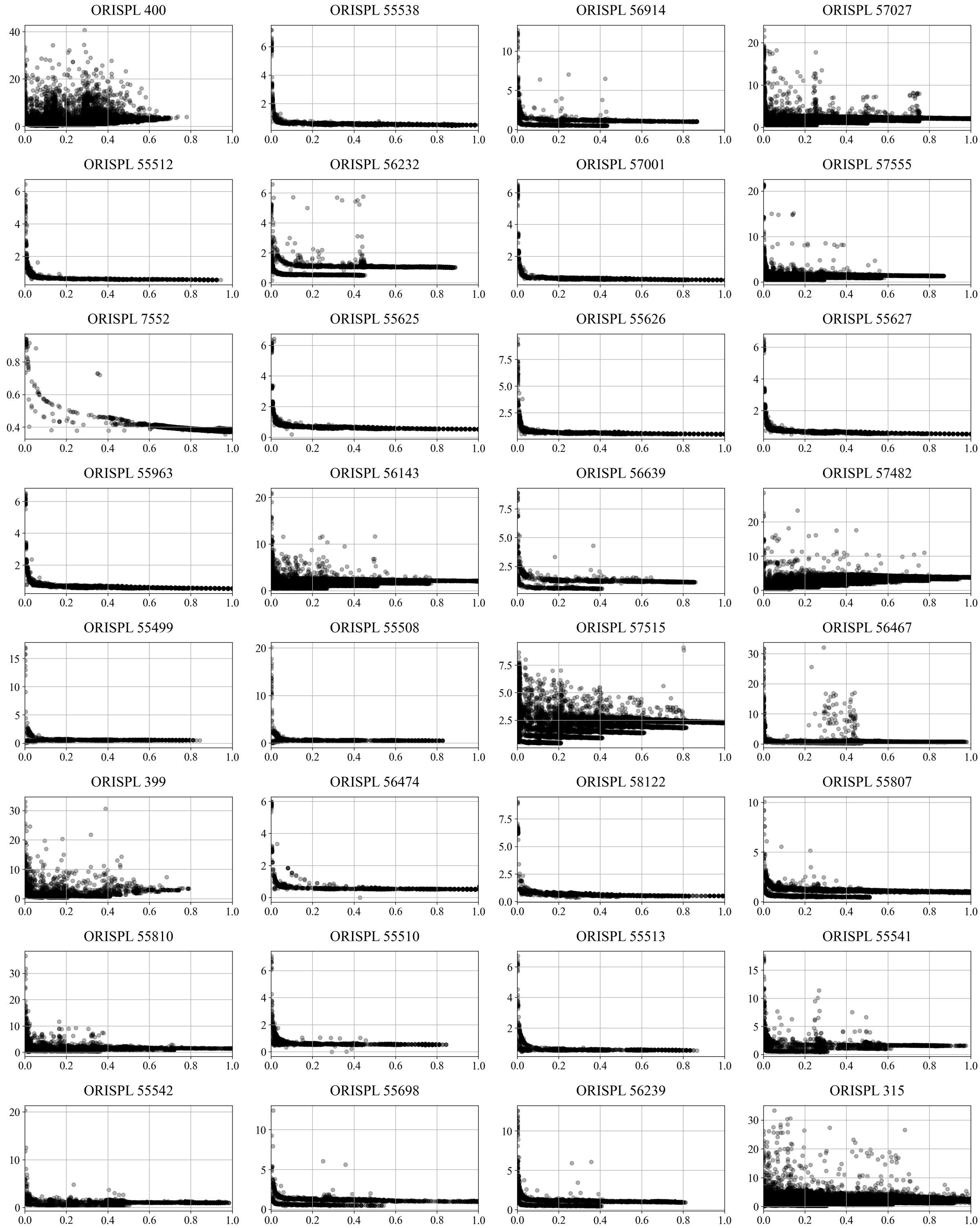

Supplementary Figure 4: Relationship between emissions intensity and capacity factor for natural gas power plants in the California Independent System Operator (CAISO) region. Each plant is identified by its unique Office of Regulatory Information Systems Plant Location (ORISPL) code, assigned by the U.S. Energy Information Administration (EIA).

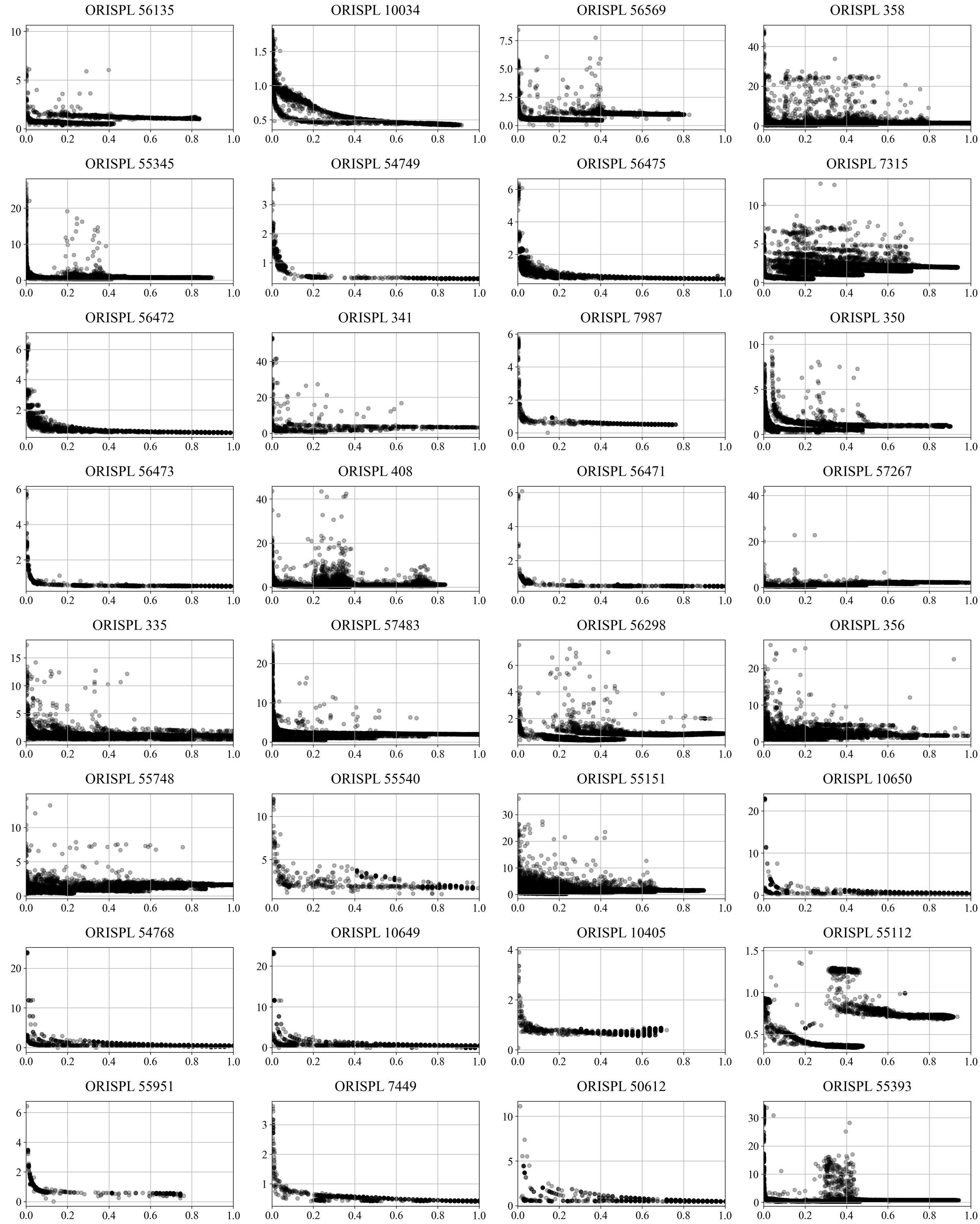

Supplementary Figure 5: Emissions intensity as a function of capacity factor for natural gas power plants in the California Independent System Operator (CAISO) region. Each plant is identified by its unique Office of Regulatory Information Systems Plant Location (ORISPL) code, assigned by the U.S. Energy Information Administration (EIA).

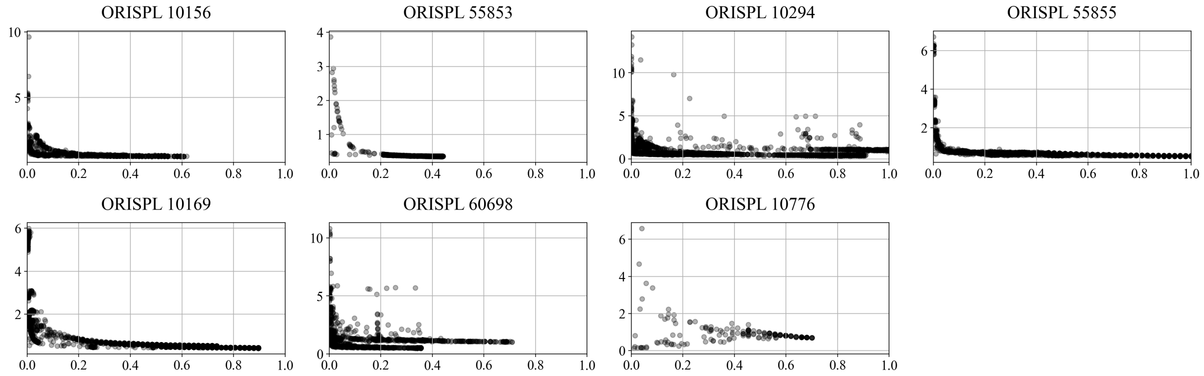

Supplementary Figure 6: Capacity factor versus emissions intensity for natural gas power plants in the California Independent System Operator (CAISO) region. Each plant is identified by its unique Office of Regulatory Information Systems Plant Location (ORISPL) code, assigned by the U.S. Energy Information Administration (EIA).

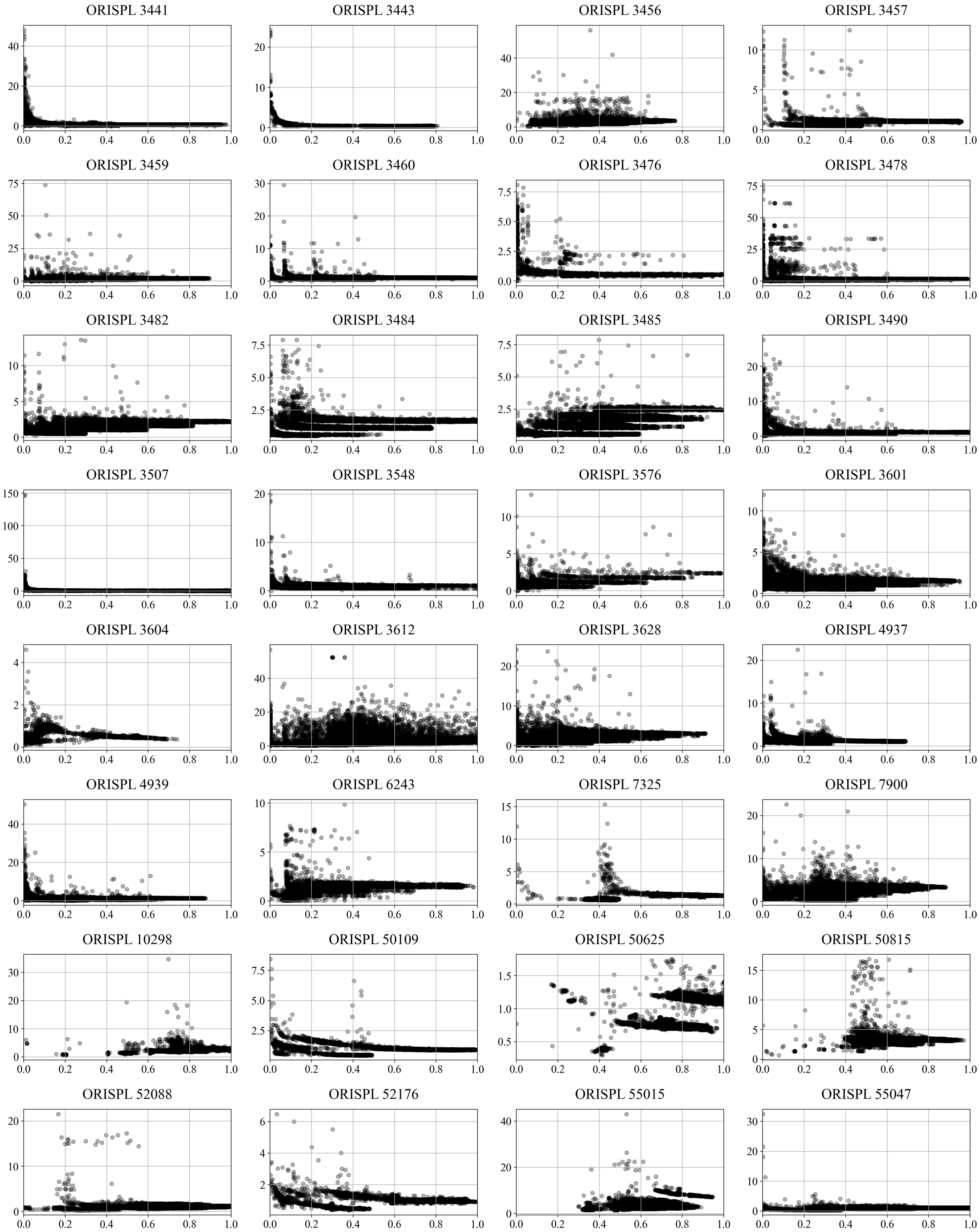

Supplementary Figure 7: Relationship between emissions intensity and capacity factor for natural gas power plants in the Electric Reliability Council of Texas (ERCOT) region. Each plant is identified by its unique Office of Regulatory Information Systems Plant Location (ORISPL) code, assigned by the U.S. Energy Information Administration (EIA).

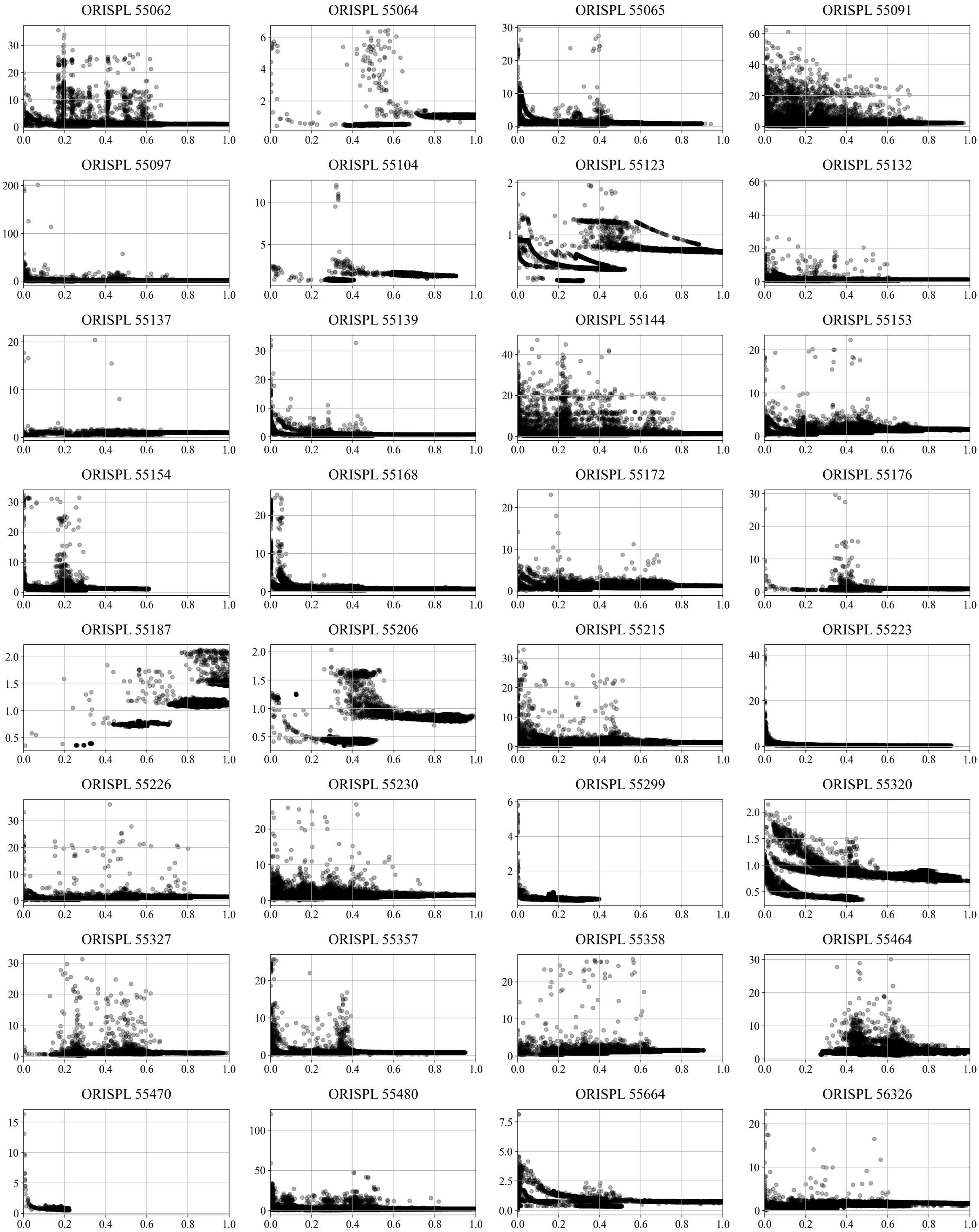

Supplementary Figure 8: Emissions intensity as a function of capacity factor for natural gas power plants in the Electric Reliability Council of Texas (ERCOT) region. Each plant is identified by its unique Office of Regulatory Information Systems Plant Location (ORISPL) code, assigned by the U.S. Energy Information Administration (EIA).

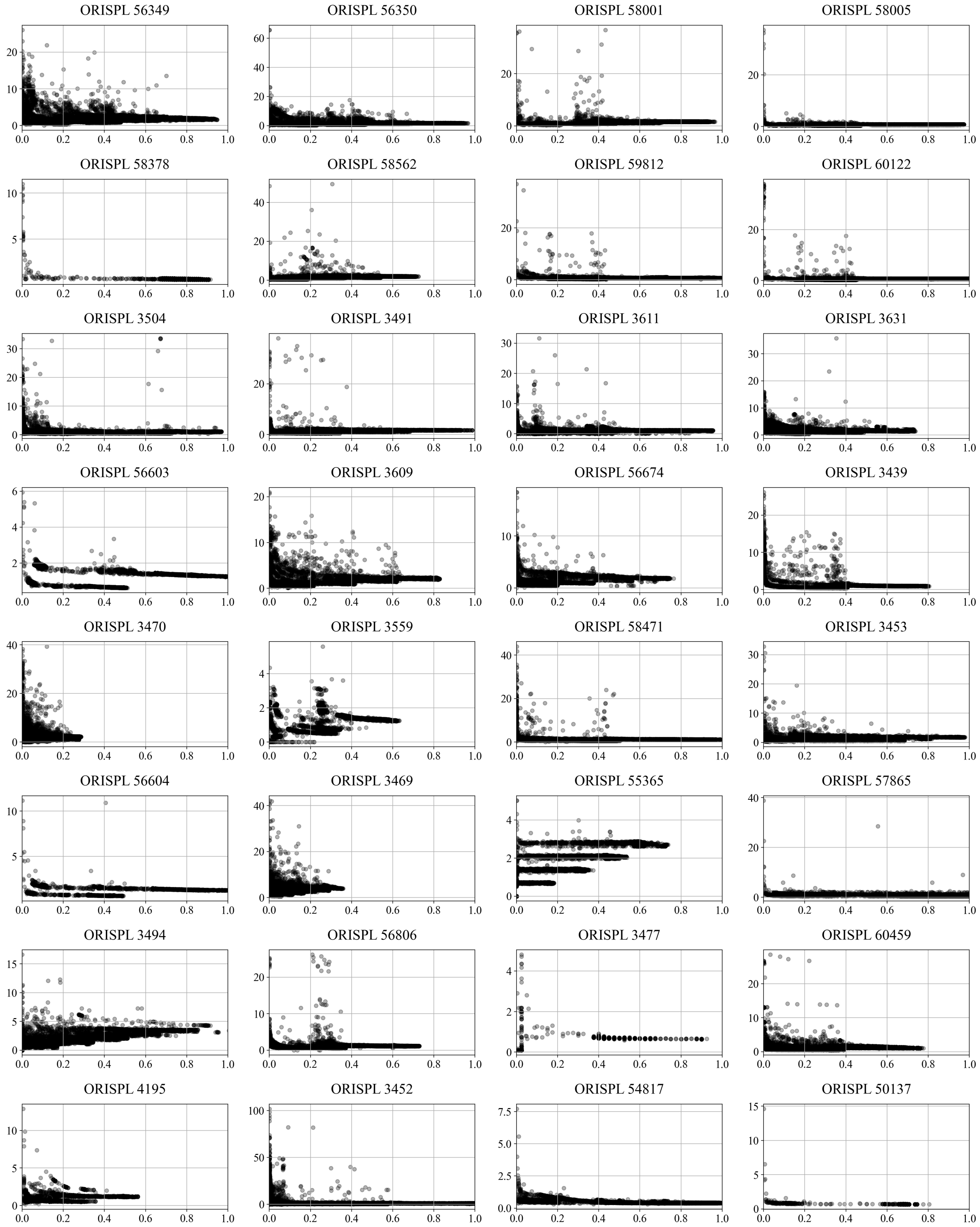

Supplementary Figure 9: Capacity factor versus emissions intensity for natural gas power plants in the Electric Reliability Council of Texas (ERCOT) region. Each plant is identified by its unique Office of Regulatory Information Systems Plant Location (ORISPL) code, assigned by the U.S. Energy Information Administration (EIA).

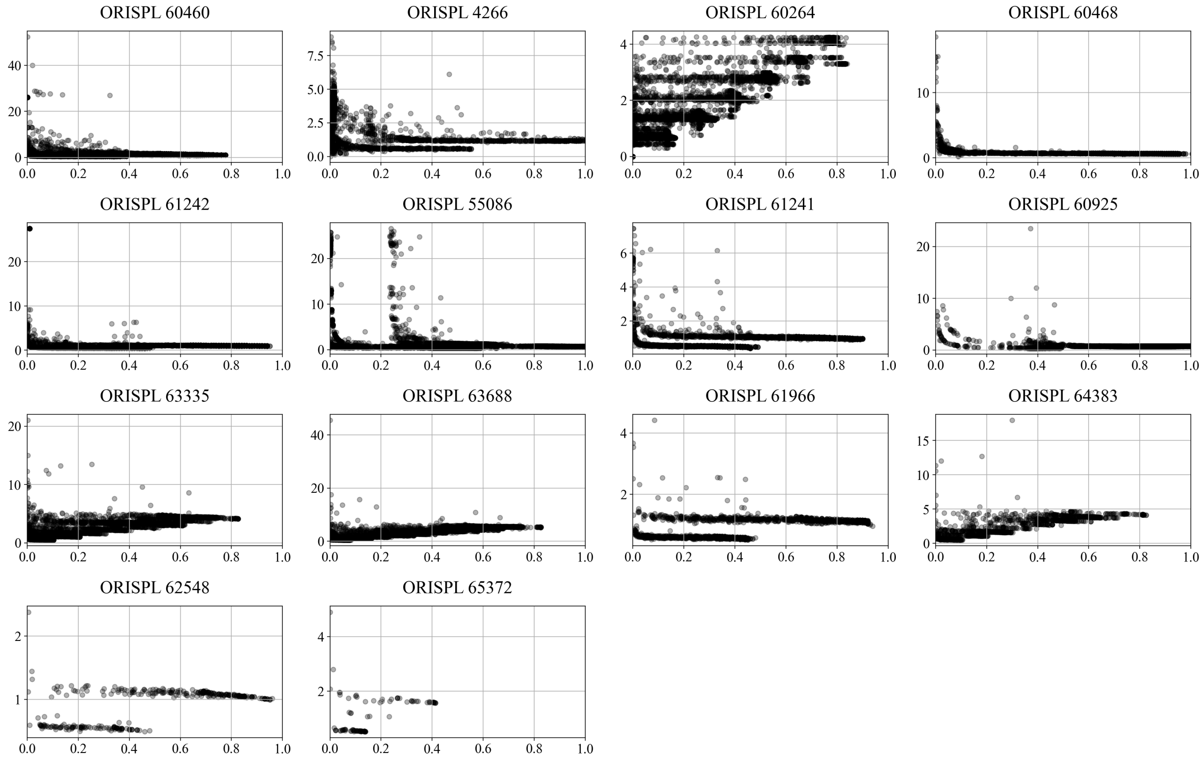

Supplementary Figure 10: Relationship between emissions intensity and capacity factor for natural gas power plants in the Electric Reliability Council of Texas (ERCOT) region. Each plant is identified by its unique Office of Regulatory Information Systems Plant Location (ORISPL) code, assigned by the U.S. Energy Information Administration (EIA).

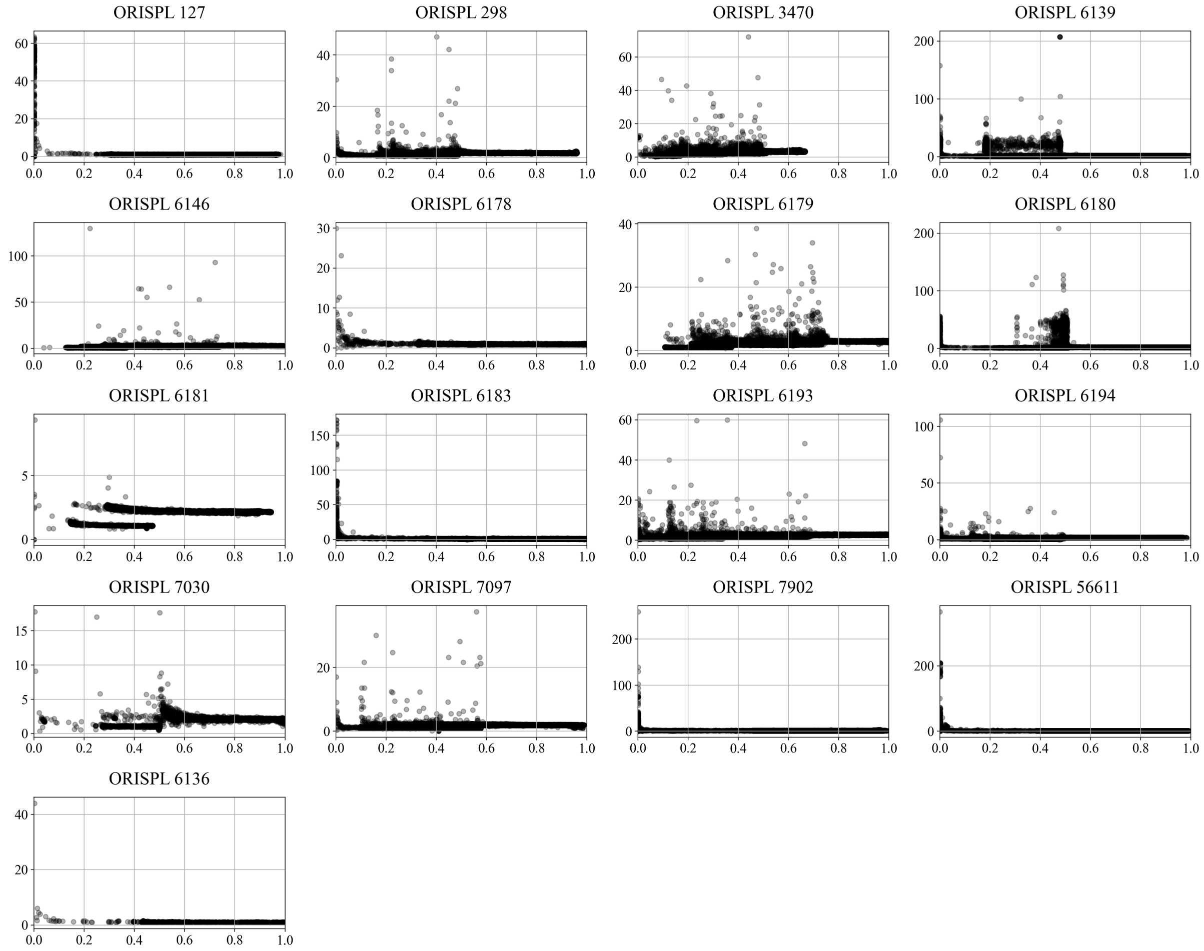

Supplementary Figure 11: Relationship between emissions intensity and capacity factor for coal-fired power plants in the Electric Reliability Council of Texas (ERCOT) region. Each plant is identified by its unique Office of Regulatory Information Systems Plant Location (ORISPL) code, assigned by the U.S. Energy Information Administration (EIA).

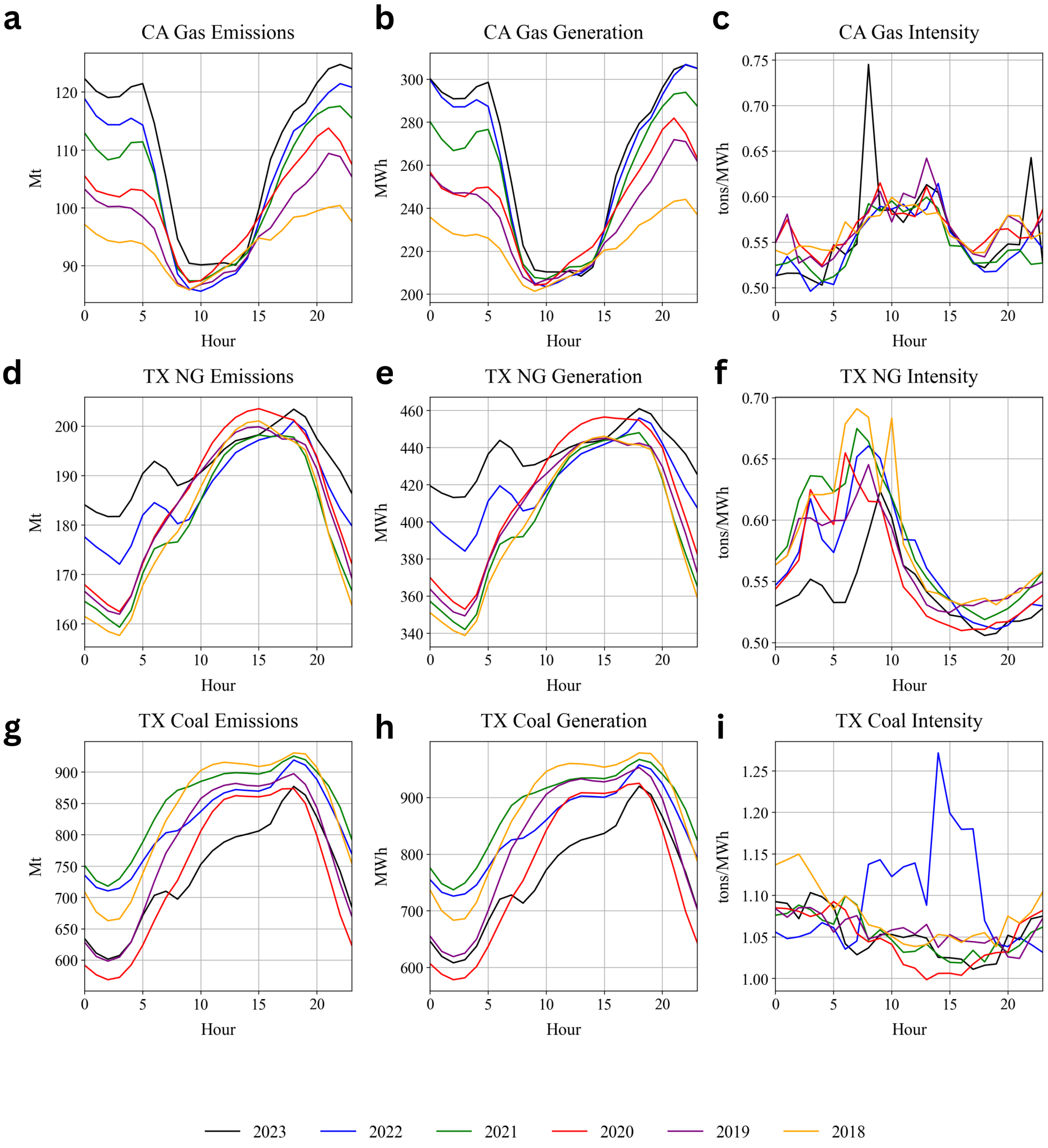

Supplementary Figure 12: Hourly variation in aggregated emissions, generation, and emissions intensity for thermal power plants in CAISO and ERCOT from 2018 to 2023. Each panel presents average values across years, with colors corresponding to different years as indicated in the legend. (a–c) Natural gas plants in the California Independent System Operator (CAISO). (d–f) Natural gas plants in the Electric Reliability Council of Texas (ERCOT). (g–i) Coal plants in ERCOT. The first column (a, d, g) shows total CO_2 emissions (megatons, Mt) by hour of the day, the second column (b, e, h) shows total electricity generation (megawatt-hours, MWh), and the third column (c, f, i) shows emissions intensity (tons of CO_2 per MWh). The data reveal distinct temporal trends, with morning and evening peaks in emissions and generation. Emissions intensity varies more substantially for coal plants than for natural gas plants, particularly in ERCOT, suggesting operational inefficiencies under varying load conditions.

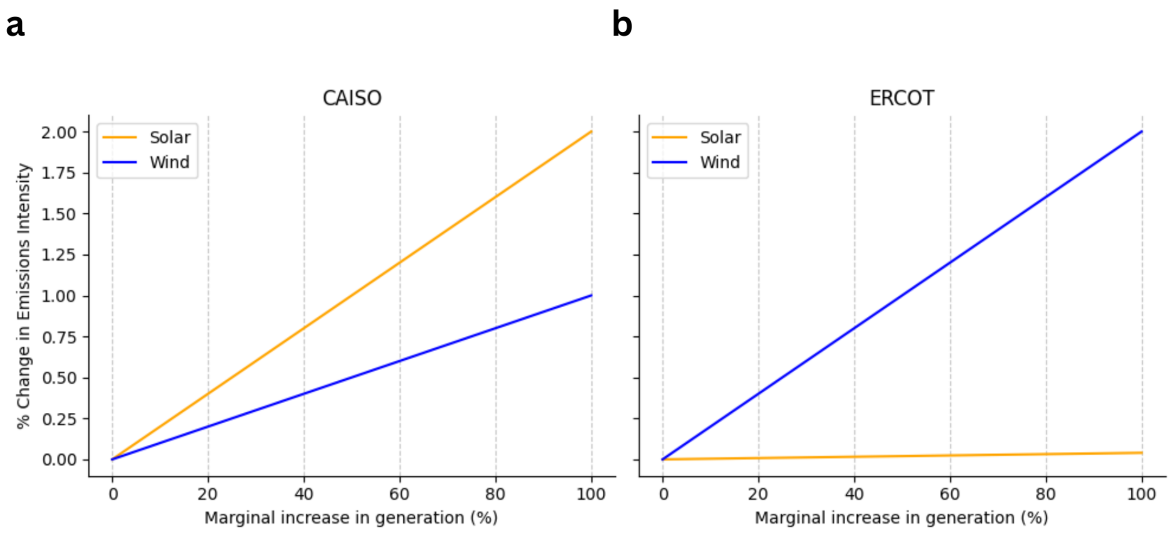

Supplementary Figure 13: Change in CO_2 emissions intensity of thermal power plants under increasing levels of renewable generation. (a) California Independent System Operator (CAISO); (b) Electric Reliability Council of Texas (ERCOT). Each panel shows the percent change in average emissions intensity (tons of CO_2 per megawatt-hour) of thermal power plants—specifically natural gas and coal units—as the system experiences a 0–100% increase in marginal generation from solar and wind, respectively. The horizontal axis represents the modeled increase in renewable generation as a percentage of historically observed hourly values, while the vertical axis indicates the corresponding percent change in emissions intensity relative to baseline conditions. As renewable generation increases, thermal plants are dispatched less frequently or operated at lower capacity factors, which can lead to elevated emissions intensity due to reduced efficiency during partial-load operation.

Supplementary Tables

Supplementary Table 1: Coefficients for the panel regression formulation for natural gas plants in CAISO for various model specifications. Significance levels: *** $p < 0.001$, ** $p < 0.01$, * $p < 0.05$.

Coefficient	M1	M2	M3	M4	M5	M6	M7	M8	M9
$\ln D'_t$	2.01^{***}	-	1.95^{***}	2.02^{***}	2.06^{***}	-	2.05^{***}	1.85^{***}	1.96^{***}
	(0.04)	-	(0.04)	(0.04)	(0.04)	-	(0.04)	(0.04)	(0.05)
$\ln S_t$	-0.27^{***}	-0.06^{**}	-	-0.24^{***}	-0.27^{***}	-0.14^{***}	-0.28^{***}	-0.24^{***}	-0.26^{***}
	(0.03)	(0.03)	-	(0.03)	(0.02)	(0.03)	(0.03)	(0.03)	(0.03)
$\ln W_t$	-0.23^{***}	-0.23^{***}	-0.22^{***}	-	-0.23^{***}	-0.13^{***}	-0.16^{***}	-0.24^{***}	-0.22^{***}
	(0.01)	(0.01)	(0.01)	-	(0.01)	(0.01)	(0.01)	(0.01)	(0.01)
$\ln S_{\text{ext},t}$	0.01	-0.15^{***}	-0.19^{***}	0.02	-	0.02	0.01	-0.07^{**}	0.00
	(0.03)	(0.03)	(0.02)	(0.03)	-	(0.03)	(0.03)	(0.03)	(0.03)
$\ln W_{\text{ext},t}$	-0.01^{**}	-0.06^{***}	-0.01^{***}	-0.03^{***}	-	-0.06^{***}	-0.01^{*}	-0.02^{***}	-0.01^{**}
	(0.00)	(0.00)	(0.00)	(0.00)	-	(0.00)	(0.00)	(0.00)	(0.00)
$\ln D_{\text{ext},t}$	0.05	0.65^{***}	0.10^{***}	0.04	-	0.17^{***}	0.04	0.09^{***}	0.08^{**}
	(0.03)	(0.03)	(0.03)	(0.03)	-	(0.03)	(0.03)	(0.03)	(0.03)
$\ln W_{\text{ramp},t}$	0.12^{***}	0.20^{***}	0.12^{***}	-0.09^{***}	0.12^{***}	0.11^{***}	-	0.14^{***}	0.12^{***}
	(0.01)	(0.01)	(0.01)	(0.01)	(0.01)	(0.01)	-	(0.01)	(0.02)
$\ln G_t$	-	-	-	-	-	2.29^{***}	-	-	-
	-	-	-	-	-	(0.06)	-	-	-
R-squared	0.84	0.83	0.84	0.84	0.84	0.84	0.84	0.84	0.82
Time FE	Y	Y	Y	Y	Y	Y	Y	N	Y
Interaction	Y	Y	Y	Y	Y	Y	Y	Y	N

Supplementary Table 2: Coefficients for the panel regression formulation for natural gas plants in ERCOT for various model specifications. Significance levels: *** $p < 0.001$, ** $p < 0.01$, * $p < 0.05$.

Coefficient	M1	M2	M3	M4	M5	M6	M7	M8	M9
$\ln D'_t$	1.88^{***}	-	1.87^{***}	1.44^{***}	1.91^{***}	-	1.83^{***}	1.75^{***}	1.76^{***}
	(0.04)	-	(0.04)	(0.04)	(0.03)	-	(0.04)	(0.04)	(0.04)
$\ln S_t$	-0.03^{***}	-0.00	-	0.07^{***}	-0.03^{***}	-0.03^{***}	0.02^{**}	-0.07^{***}	-0.03^{***}
	(0.01)	(0.01)	-	(0.01)	(0.01)	(0.01)	(0.01)	(0.00)	(0.01)
$\ln W_t$	-0.34^{***}	-0.26^{***}	-0.33^{***}	-	-0.35^{***}	-0.33^{***}	-0.30^{***}	-0.34^{***}	-0.32^{***}
	(0.01)	(0.01)	(0.01)	-	(0.01)	(0.01)	(0.01)	(0.01)	(0.01)
$\ln S_{\text{ext},t}$	0.01^{*}	-0.00	-0.00	0.02^{**}	-	0.01^{*}	0.00	0.04^{***}	0.01^{*}
	(0.01)	(0.01)	(0.01)	(0.01)	-	(0.01)	(0.01)	(0.01)	(0.01)
$\ln W_{\text{ext},t}$	-0.02^{***}	-0.09^{***}	-0.02^{***}	-0.24^{***}	-	-0.03^{***}	-0.03^{***}	-0.03^{***}	-0.02^{***}
	(0.01)	(0.01)	(0.01)	(0.01)	-	(0.01)	(0.01)	(0.01)	(0.01)
$\ln D_{\text{ext},t}$	0.02	1.39^{***}	0.03	0.29^{***}	-	0.02	0.03	-0.02	0.05
	(0.05)	(0.03)	(0.05)	(0.05)	-	(0.04)	(0.05)	(0.04)	(0.05)
$\ln W_{\text{ramp},t}$	0.12^{***}	0.08^{***}	0.11^{***}	-0.02^{***}	0.12^{***}	0.12^{***}	-	0.14^{***}	0.12^{***}
	(0.01)	(0.01)	(0.01)	(0.01)	(0.01)	(0.01)	-	(0.01)	(0.01)
$\ln D_t$	-	-	-	-	-	1.89^{***}	-	-	-
	-	-	-	-	-	(0.04)	-	-	-
R-squared	0.78	0.77	0.78	0.77	0.78	0.78	0.78	0.78	0.75
Time FE	Y	Y	Y	Y	Y	Y	Y	Y	Y
Interaction	Y	Y	Y	Y	Y	Y	Y	Y	Y

Supplementary Note 1

We test several specifications of the plant and time fixed effects model. Model coefficients, standard errors, R-squared values and key parameters are summarized in Supplementary Tables 1 and 2. The original structure of the fixed-effects specification was derived from Bushnell and Wolfram[1] and Graf et al.[2] The former developed a logarithmic formulation to attribute the effect of fuel consumption, unit age, ambient temperature on power plant heat rate and fuel efficiency using entity and time fixed-effects. Graf et al.[2] use a similar logarithmic formulation and fixed-effects structure as Bushnell and Wolfram[1], except with different independent variables and applied to annual power plant data. In Graf et al.,[2] the authors aggregate generation from solar and wind into a single term, R_t, and the control variables include the nameplate capacity and share of gas production.

In developing our main formulation, we deviate from Graf et al.,[2] in that we disaggregate generation from solar and wind into separate independent variables. In addition, we consider several control variables outlined in the methods. Lastly, instead of using residual demand as an independent variable, we use residual thermal generation to account for the non-stationary generation requirements in daily dispatch processes. This enables us to characterize the residual effect on thermal generators as a whole and control for temperature-dependent variability in demand.

Looking at the coefficients of the alternate regression models for CAISO in Supplementary Table 1, we see that there is limited variance in the coefficients for solar and wind. When demand is omitted in Supplementary Equation (2), the model attributes additional generation from thermal plants to solar generation and demand in CAISO's trading partners. Similarly, when we consider net demand in place of residual thermal generation in Supplementary Equation (6), the model coefficients for external demand and wind generation are more pronounced.

Similarly, on examining the coefficients for the regression models for ERCOT in Supplementary Table 2, the coefficient for wind generation is disparate in two alternate specifications - when demand is used in place of residual thermal generation in M6 and when wind ramp is omitted in Supplementary Equation (7). In Supplementary Equation (6), the coefficient for both solar and wind is smaller, while that of external demand is more than three times higher. In Supplementary Equation (7), omitting wind intermittency lowers the coefficient magnitude given that the effect of intermittency is to increase the generation by thermal plants in other models.

Main specification

M1: $$\ln y_t = \alpha + \beta_1 \ln D'_t + \beta_2 \ln S_t + \beta_3 \ln W_t + \beta_4 \ln \overline{W}_t + \gamma \ln \mathbf{X}_t + \eta_{t,m} + \eta_{t,y} + \epsilon \quad (1)$$

Exclude residual demand

M2: $$\ln y_t = \alpha + \beta_2 \ln S_t + \beta_3 \ln W_t + \beta_4 \ln \overline{W}_t + \gamma \ln \mathbf{X}_t + \eta_{t,m} + \eta_{t,y} + \epsilon \quad (2)$$

Exclude solar generation

M3: $$\ln y_t = \alpha + \beta_1 \ln D'_t + \beta_3 \ln W_t + \beta_4 \ln \overline{W}_t + \gamma \ln \mathbf{X}_t + \eta_{t,m} + \eta_{t,y} + \epsilon \quad (3)$$

Exclude wind generation

M4: $$\ln y_t = \alpha + \beta_1 \ln D'_t + \beta_2 \ln S_t + \beta_4 \ln \overline{W}_t + \gamma \ln \mathbf{X}_t + \eta_{t,m} + \eta_{t,y} + \epsilon \quad (4)$$

Exclude external control variables

M5: $$\ln y_t = \alpha + \beta_1 \ln D'_t + \beta_2 \ln S_t + \beta_3 \ln W_t + \beta_4 \ln \overline{W}_t + \eta_{t,m} + \eta_{t,y} + \epsilon \quad (5)$$

Alternate treatment of demand

M6: $$\ln y_t = \alpha + \beta_1 \ln D_t + \beta_2 \ln S_t + \beta_3 \ln W_t + \beta_4 \ln \overline{W}_t + \gamma \ln \mathbf{X}_t + \eta_{t,m} + \eta_{t,y} + \epsilon \quad (6)$$

Exclude wind ramp

M7: $$\ln y_t = \alpha + \beta_1 \ln D'_t + \beta_2 \ln S_t + \beta_3 \ln W_t + \gamma \ln \mathbf{X}_t + \eta_{t,m} + \eta_{t,y} + \epsilon \quad (7)$$

Exclude time fixed effects

M8: $$\ln y_t = \alpha + \beta_1 \ln D'_t + \beta_2 \ln S_t + \beta_3 \ln W_t + \beta_4 \ln \overline{W}_t + \gamma \ln \mathbf{X}_t + \epsilon \quad (8)$$

Exclude interaction terms

M9: $$\ln y_t = \alpha + \beta_1 \ln D'_t + \beta_2 \ln S_t + \beta_3 \ln W_t + \beta_4 \ln \overline{W}_t + \gamma \ln \mathbf{X}_t + \eta_{t,m} + \eta_{t,y} + \epsilon \quad (9)$$

Supplementary references

[1] James B Bushnell and Catherine Wolfram. Ownership change, incentives and plant efficiency: The divestiture of us electric generation plants. 2005.

[2] Christoph Graf and Claudio Marcantonini. Renewable energy and its impact on thermal generation. *Energy economics*, 66:421–430, 2017.
